# Supplementary material for: Content-rich biological network constructed by mining PubMed abstracts
Source: BMC Bioinformatics. 2004 Oct 8;5:147. doi: 10.1186/1471-2105-5-147 (PMC528731; doi:10.1186/1471-2105-5-147)
Supplement: Additional File 5 — The original Chilibot query results of the term "long-term potentiation (LTP)" and 22 other terms, limiting the latest references analyzed to the years 1990, 1995, 2000, and 2004. [file 1471-2105-5-147-S5.bz2 › chilibotAdditionalFile5/ltp2000/html/PKC_CAMKIV.html]

 


 **PKC** and **CAMKIV** 
  
Found 1 abstracts in PubMed,  **1 abstracts were retrieved and analyzed**.  


---

 Search Google  |
 PDF files only 
|  EDU domain only 

---

- Biochim Biophys Acta, 1999   **Transcriptional regulation of a receptor protein tyrosine phosphatase gene hPTP J by **PKC** mediated signaling pathways in Jurkat and Molt 4 T lymphoma cells.** .
  The recently cloned type II receptor protein tyrosine phosphatase RPTP gene hPTP J is a new member of the MAM meprin, A5, PTPmicro domain subfamily.
  We previously reported that hPTP J mRNA was detected significantly in Jurkat T lymphoma cells and its expression was completely down regulated by phorbol myristate acetate PMA .
  In this study, we investigated what signaling pathways molecules are involved in the transcriptional regulation of hPTP J expression in Jurkat and Molt 4 T cell lines.
  The hPTP J transcription was transiently up regulated 20 min after the addition of PMA 20 ng ml to the Jurkat culture, followed by the complete down regulation in 8 h after PMA addition.
  The transient up regulation and the complete down regulation induced by PMA was blocked by a **PKC** specific inhibitor, GF109203X, suggesting that the regulatory effect of PMA on the hPTP J transcription depends on protein kinase C activation.
  hPTP J transcription was down regulated not only by PMA but also by several signaling modulators including 1 oleoyl 2 acetylglycerol, forskolin, orthovanadate, manumycin and okadaic acid.
  Therefore, several signaling molecules such as protein tyrosine phosphatases, PP2A **CaMKIV** and Ras are required for hPTP J transcription in Jurkat and Molt 4 cells.
